# Ethnic and racial-specific differences in levels of centrosome-associated mitotic kinases, proliferative and epithelial-to-mesenchymal markers in breast cancers

**DOI:** 10.1186/s13008-022-00082-3

**Published:** 2022-12-09

**Authors:** Yainyrette Rivera-Rivera, Geraldine Vargas, Neha Jaiswal, Angel Núñez-Marrero, Jiannong Li, Dung-Tsa Chen, Steven Eschrich, Marilin Rosa, Joseph O. Johnson, Julie Dutil, Srikumar P. Chellappan, Harold I. Saavedra

**Affiliations:** 1 Pharmacology and Cancer Biology Division, Department of Basic Sciences, Ponce Research Institute, Ponce Health Sciences University, 7004, Ponce, PR 00716-2347 USA; 2 Roswell Park Comprehensive Cancer Center, Buffalo, NY USA; 3 Biochemistry and Cancer Biology Division, Ponce Research Institute, Ponce Health Sciences University, Ponce, PR USA; 4 Department of Biostatistics and Bioinformatics, Moffitt Cancer Center, Tampa, FL USA; 5 Departments of Anatomic Pathology, Moffitt Cancer Center, Tampa, FL USA; 6 Analytic Microscopy Core, Moffitt Cancer Center, Tampa, FL USA; 7 Department of Tumor Biology, H. Lee Moffitt Cancer Center and Research Institute, Tampa, FL USA

**Keywords:** TTK, TBK1, Nek2, Vimentin, E-cadherin, Ki67, Tissue microarray, Breast cancer

## Abstract

Molecular epidemiology evidence indicates racial and ethnic differences in the aggressiveness and survival of breast cancer. Hispanics/Latinas (H/Ls) and non-Hispanic Black women (NHB) are at higher risk of breast cancer (BC)-related death relative to non-Hispanic white (NHW) women in part because they are diagnosed with hormone receptor-negative (HR) subtype and at higher stages. Since the cell cycle is one of the most commonly deregulated cellular processes in cancer, we propose that the mitotic kinases TTK (or Mps1), TBK1, and Nek2 could be novel targets to prevent breast cancer progression among NHBs and H/Ls. In this study, we calculated levels of TTK, p-TBK1, epithelial (E-cadherin), mesenchymal (Vimentin), and proliferation (Ki67) markers through immunohistochemical (IHC) staining of breast cancer tissue microarrays (TMAs) that includes samples from 6 regions in the Southeast of the United States and Puerto Rico -regions enriched with NHB and H/L breast cancer patients. IHC analysis showed that TTK, Ki67, and Vimentin were significantly expressed in triple-negative (TNBC) tumors relative to other subtypes, while E-cadherin showed decreased expression. TTK correlated with all of the clinical variables but p-TBK1 did not correlate with any of them. TCGA analysis revealed that the mRNA levels of multiple mitotic kinases, including *TTK, Nek2, Plk1, Bub1,* and *Aurora kinases A and B,* and transcription factors that are known to control the expression of these kinases (e.g. *FoxM1 and E2F1-3*) were upregulated in NHBs versus NHWs and correlated with higher aneuploidy indexes in NHB, suggesting that these mitotic kinases may be future novel targets for breast cancer treatment in NHB women.

**Supplementary Information:**

The online version contains supplementary material available at 10.1186/s13008-022-00082-3.

## Introduction

Breast cancer is the most commonly diagnosed cancer worldwide, representing 12% of all new annual cancer cases globally [1]. In America, breast cancer is the most commonly diagnosed cancer among women, and in the United States (US), it is the second leading cause of all cancer mortality among women, after lung cancer. Molecular epidemiology studies provide evidence for racial/ethnic differences in the aggressiveness and survival of breast cancer patients revealing that non-Hispanic black (NHB) women and Hispanic/Latino women (H/L) have poorer outcomes and survival compared with non-Hispanic White (NHW) women [2–6]. H/L women from the Caribbean (C-H/L: from Puerto Rico, Dominican Republic, and Cuba) have higher incidences [7, 8] and poorer survival outcomes than other H/L [9, 10]. Thus, compelling evidence demonstrates the disparities among NHB and H/L who are more likely to be diagnosed at later stages (stages II-IV) compared with NHW women [4, 11–16]. NHB and H/L also have a higher probability of being diagnosed with triple-negative breast cancer (TNBC), a subtype that lacks hormone receptors and for which there are no effective biological treatments. H/L and NHB women with TNBC have significantly poorer 5 year survival outcomes than NHW women with TNBC [17]. While TNBC and socioeconomic status independently contribute to poor outcomes in H/L and NHB women with breast cancer, worse survival rates occur even after adjusting for socioeconomic status, estrogen receptor (ER) and progesterone receptor (PR) status, and access to health care [4]. This is supported by observations that NHB women have higher mortality rates than NHW women, even when detected with the Luminal (ER + PR + and Her2 + or –) subtypes, which have the best prognostic outcomes of any breast cancer subtype [18–20]. The same trend is seen in black H/L women with breast cancer, who have lower survival percentages than their white counterparts [21]. Thus, novel therapeutic strategies are needed to reduce the high mortality rates of H/L and NHB women with advanced breast cancer. Elucidating the expression patterns of centrosome-associated mitotic kinases in specific racial/ethnic groups and therapies against these kinases has the potential to greatly improve the survival outcomes of H/L and NHB women with breast tumors.

Over the past decade, many studies have shown a direct role of protein kinase dysregulation (through protein overexpression or mutations) in several human diseases including cancer [22]. Kinases are common drug targets for cancer treatment [23], and therapies against mitotic kinases are an emerging strategy against cancer [24]. TTK is a kinase that initiates the spindle assembly checkpoint (SAC), a mechanism that prevents the missegregation of chromosomes by ensuring the attachment of kinetochores to the spindle at metaphase [25–27]. TTK recruits several regulators of the SAC, including KNL1, MAD1, and BUB1 kinases into centromeres, and is an essential protein in cancer cells [28]. TBK1, an IKK (IκB Kinase)-related kinase that mediates inflammatory responses [29] and that is involved in the stabilization of microtubules [30] is also an important regulator of the SAC in breast and lung cancer [31]. NIMA-related kinase 2 (Nek2) triggers centrosome separation at the G2 phase and the SAC [32–34]. When dysregulated these kinases modulate rates of centrosome amplification-driven chromosome instability (CA/CIN) [30, 35–41], which is an abnormal process that promotes tumor initiation and cell invasion [42–44]. Therefore, chemical inhibitors against these kinases prevent the further generation of CA/CIN but also lead to massive chromosome losses that result in cell death [24, 45–47].

Recent publications from our laboratory and others have found that centrosome-associated mitotic kinases are involved in early intermediate steps to metastasis, including cell migration and invasion (reviewed in [24, 48]. Our group has demonstrated that the pharmacological or genomic silencing of TTK suppressed the epithelial-to-mesenchymal transition (EMT) and invasion of mesenchymal, TNBC cells through different mechanisms including the induction of the transcription factor KLF5, the induction of mi-RNA (miR) 200, the decreased expression of the EMT-associated miR-21, and the suppression of TGF-β-induced SMAD-3 phosphorylation [49]. We also demonstrated that Nek2 drives the EMT of TNBC cell lines by regulating EMT markers including E-cadherin, and Vimentin, as well as the EMT transcription factors Slug and Zeb1 [35]. To further explore these findings, here we present data on the expression of *TTK*, *Nek2*, and *TBK1* mRNA in different ethnic and racial groups. In addition, we present data on expression levels of TTK and TBK1 and how that correlates with EMT markers including E-cadherin and Vimentin, using a novel set of tissue microarrays (TMAs) with invasive ductal carcinoma (IDC) breast tissue samples from NHB, C-H/L, and NHW women from Southeast USA and Puerto Rico. These TMAs were previously designed and constructed as part of an effort between Ponce Health Sciences University-Ponce Research Institute and Moffitt Cancer Center with the purpose to study drivers of breast cancers in different ethnic and racial populations [50]. Thus, our findings will provide novel knowledge related to future strategies for therapeutic intervention in NHBs and H/Ls with advanced stages of breast cancer.

## Materials and methods

### Ethics and consent statement

The protocol for this study was classified as exempt by the Institutional Review Board from the Ponce Health Sciences University (Human subjects assurance number FWA00000345) and approved under protocol number 160115-HS.

### Bioinformatic analysis of gene expression

Gene expression in breast tumors was done using cBIOPORTAL [51, 52] analyses by using either the METABRIC or the TCGA databases. Overexpression is defined as a z-score threshold ± 2.0 of gene expression in tumors relative to diploid samples. Filters were applied, including subtype (both databases), race, and ethnicity (only available in TCGA). Data was downloaded onto Excell and analyzed using statistics (below).

### Patient cohorts from the breast cancer tissue microarray (TMA)

For this study, we used formalin-fixed, paraffin-embedded TMAs from the National Cancer Institute (NCI) Minority Biospecimen/Biobanking Geographic Management Program for region 3 (BMaP-3), now BMaP region 2. The TMA includes breast cancer samples characterized pathologically by hematoxylin/eosin (H&E) and immuno-histochemically with antibodies recognizing ER, PR, and Her2 [50]. The breast cancer samples were collected in a de-identified fashion by six academic centers (Moffitt Cancer Center, Emory Winship Cancer Center, Ponce Health Sciences University, Tulane University, the University of Alabama at Birmingham, and the University of Mississippi Medical Center) serving the Southeast of the United States and Puerto Rico. Samples are from patients older than 18 years of age and primary, invasive ductal carcinomas (no metastasis). The subtypes included in the array are TNBC (Her2-ER-PR-), Luminal (Her2 − ER + PR + or Her2 + ER + PR +), and Her2 + (Her2 + ER-PR-). The TMA includes breast cancer tissues from 147 NHB, 168 H/L, and 112 NHW women subtyped into TNBC (n = 85), Her2 + (n = 26), and Luminal (n = 119). Survival outcomes or genotyping for determining genetic ancestry were not allowed for samples from some of the institutions. Some archival samples were not subtyped with ER, PR, or Her2. Another major limitation is that the TMA does not contain enough patients from different ethnicities and races to make statistically sound conclusions about expression within pathological subtypes in specific races (e.g. NHB vs. NHW) or ethnicities (non-H/L vs. H/L). A sample size of at least 100 tumors per ethnic/racial category or subtype would be needed to achieve an 85% power to detect the difference (50% versus 20%) using Fisher exact test and a two-sided 5% Type I error. A minor technical limitation is that very rarely were cores lifted and were lost during the staining procedure and thus the number of samples for each marker for each race and ethnicity slightly varies.

### Immunohistochemistry and scoring

Tissue slides were de-paraffinized in xylene and rehydrated in 100%, 95%, 80%, and 75% ethanol followed by a distilled H_2_O wash. Endogenous peroxidase was blocked with 3% hydrogen peroxide for 15 min at room temperature. Incubation of 40 min for antigen retrieval was done using a citrate antigen retrieval solution that was preheated to 95 ℃ and then allowed to cool down for 20 min. Tissues were then incubated in primary antibody for TTK (1:100) (Cell Signal #5469S), E-cadherin (1:300) (Cell Signal #3195S), Vimentin (1:200) (Cell Signal #5741S), and Ki67 (1:200) (Cell Signal #9449S) (or PBS for negative controls) overnight at 4–8 ℃ in a sealed humid chamber. Tissues were then incubated with a biotin-conjugated secondary antibody solution for 30 min in the humid chamber. The secondary antibody was included in the Super Sensitive Link Label IHC kit (Cat. No. LP000-ULE, BioGenex, Fremont, CA); this same kit was used in subsequent steps for signal development following the manufacturer’s instructions. For slide mounting, tissues were dehydrated with 85%, 90%, 95%, and 100% ethanol and lastly incubated in xylene, oven-dried at 37 ℃ for 30 min, and sealed with permanent mounting medium and coverslips. Slides that were used for pathology analyses were counterstained with hematoxylin–eosin. Immuno-stained slides were independently and blindly scored for the number of positive cells by the board-certified pathologist Dr. Marilin Rosa. The whole tumor area was scored to account for tumor heterogeneity with at least four 40X visual fields per core counted. Pathologists used a 0 to 5 scoring system, in which a score of 0 meant 0% positive cells per 40X visual field, and 1, 2, 3, 4, and 5 meant less than 1%, 1–10%, 11–33%, 34–66%, and 67–100% positive cells per 40X visual field, respectively. TMA slides were digitally imaged with a Leica Aperio AT2 slide scanner where additional scoring was conducted using the Aperio’s Positive Pixel Count algorithm by Mr. Joseph Johnson at the Moffitt Cancer Center’s Imaging Core. No data points or subjects were excluded from our analyses.

### Statistical analysis

Descriptive statistics for TTK or TBK1 cohort were done using compareGroups r package. For all categorical variables frequency (percentage) is presented. The associations between categorical clinic variables and each biomarker score were evaluated using the Chi-square test when each biomarker score was treated as the categorical variable while using the Kruskal–Wallis test when the score was treated as the continuous variable. Bioinformatic statistical analysis of TCGA and/or METABRIC datasets from the cBioportal was done using a T-test (2-tails, unequal variance).

## Results

### The expression of centrosome-mitotic kinase mRNAs co-occurs with epithelial-to-mesenchymal markers

**Fig. 1:**
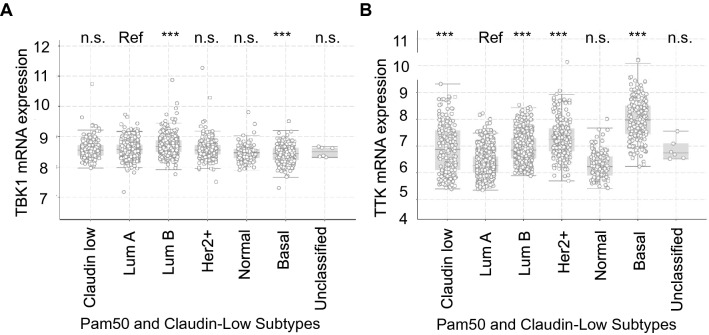
TBK1 and TTK are overexpressed in different breast cancer subtypes. METABRIC (RNA seq Z values) analysis of mRNA expression of TBK1 and TTK by breast cancer molecular subtype classification. cBioPortal analyses of mRNA expression of TBK1 and TTK mitotic kinases in lobular and ductal breast cancer subtypes determined by PAM50 molecular subtyping, n = 2509. A z-score threshold ± 2.0 was used for optimal results. Significance was addressed with ANOVA

We assessed the expression of *TTK* and *TBK1* mRNAs using the METABRIC database, using the cBioPortal for Cancer Genomics, an open-access resource for multidimensional cancer genomics datasets analysis tool [51–53]. METABRIC determined global gene expression in over 2000 patients using RNA seq; this database subtyped breast tumors into molecular subtypes (Luminal A or B, Her2 + , Basal, and Normal-like) using PAM50 analysis. The analysis was done using Luminal A breast cancer as the baseline since it is the molecular subtype with the best prognosis [19]. In contrast, basal breast cancers (over 70% of which are TN, or Her2-ER-PR-) have the worst prognosis. A previous publication from our laboratory using the same database showed significant overexpression of *Nek2* in Claudin Low, Luminal B, Her2 + , and Basal relative to Luminal A breast cancers [35]. In addition, we have published that the overexpression of *Nek2* or *TTK* mRNA is associated with poor overall- and relapse-free survival of breast cancer patients [36]. *TBK1* mRNA is significantly overexpressed in Luminal B breast cancers and significantly underexpressed in basal breast cancers (Fig. 1A). *TTK* mRNA is significantly overexpressed in Claudin-Low, Luminal B, Her2 + , and basal breast cancers (Fig. 1B).

Next, we examined the gene expression of *TTK* and *TBK1* mRNAs in different subtypes of breast cancers using TCGA (n = 996 breast cancers) [54] and METABRIC (n = 1904 breast cancers), Table 1. This analysis found more frequent upregulation of *TTK* in basal breast cancers (31.6% in TCGA and 32.2% in METABRIC) than in other subtypes. *TBK1* was overexpressed in some basal breast cancer patients (2.3% in TCGA and 2% in METABRIC), and downregulated in other basal breast cancer patients (5.3% in TCGA and 12% in METABRIC). Notably, *TBK1* is overexpressed in 26.7% of TCGA breast cancers that do not fall within the molecular classifications, or that failed to classify. The differences between data obtained through METABRIC and TCGA may include technical differences in performing the assays (for example, METABRIC data was obtained using Agilent microarray platforms and TCGA used RNA seq), as well as because they sample different populations (METABRIC includes population from the United Kingdom and Canada, while TCGA includes population from the United States of America).

**Table 1:** Percentage of breast cancer patients with dysregulated expression of TTK and TBK1

TCGA					METABRIC			
Subtype	N	% TTK over-expressed	% TBK1 over-expressed	% TBK1 under-expressed	N	% TTK over-expressed	% TBK1 over-expressed	% TBK1 under-expressed
All	996	6.6	8.0	2.2	1904	5.1	5.9	3.3
Basal	171	31.6	2.3	5.3	199	32.2	2	12.0
HER2 +	78	3.8	6.4	3.8	220	7.7	6.8	2.7
Luminal A	499	0.0	6.0	1.8	679	0.0	3.4	1.3
Luminal B	197	4.6	18.8	0	461	0.7	11.5	1.1
Normal	36	0.0	0	2.8	140	0.0	2.9	6.4
Non-classified	15	0.0	26.7	0	6	0.0	0.0	0.0
Claudin-Low	N/A	N/A	N/A	N/A	199	6.5	6.5	4.5

Gene expression of TTK, TBK1, and Nek2 mRNAs from basal breast cancers using TCGA and METABRIC datasets from cBioPortal. Analysis used n = 2509 samples from METABRIC and n = 1084 TCGA samples. A z-score threshold of ± 2.0 was used for optimal results

### The expression of centrosome-mitotic kinase mRNAs is elevated in non-Hispanic black women with breast cancer

**Fig. 2:**
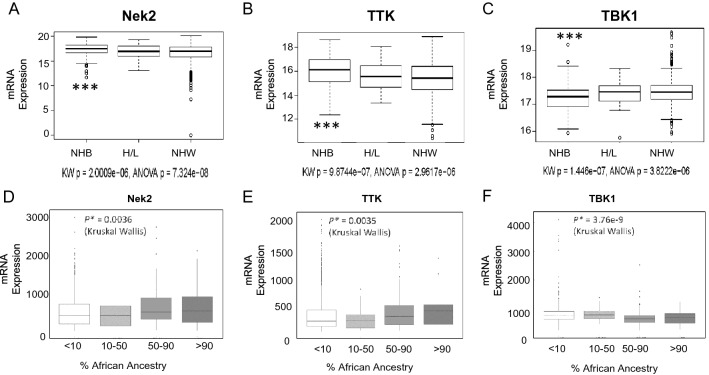
The mRNA expression of mitotic kinases in different racial and ethnic groups. Expression patterns of Nek2, TTK, and TBK1 in breast cancer patients from TCGA (**A**–**C**). A z-score threshold ± 2.0 was used for optimal results. P-values were calculated using a pairwise ANOVA t-test and a Dunn analysis for the Kruskal–Wallis Test. The results for the three kinases resulted in P < 2.96e-06 to 7.3e-08 (***). Expression of Nek2, TTK, and TBK1 as a function of ancestry. P-values were calculated using the Kruskal–Wallis Test.

We examined the TCGA dataset to understand the differences in the mRNA expression of *Nek2* (Fig. 2A), *TTK* (Fig. 2B), and *TBK1* (Fig. 2C) in NHW (n = 743), NHB (n = 187), and H/L (n = 39) populations with breast cancer based on their self-reported race and ethnicity. This analysis revealed that there were significant differences in the expression of the three kinases between NHB versus NHW, with *Nek2* and *TTK* being overexpressed and *TBK1* underexpressed in NHB (Fig. 2A–C). However, the H/L versus NHW differences were not significant, perhaps due to the limited sample size (n = 39), and it is known that TCGA did not recruit sufficient numbers of ethnic and racial minorities with breast cancer [57]. Therefore, we calculated the power to detect differences (G*Power 3.1), estimated based on the observed NHB versus NHW results for three kinases. Assuming an alpha level of 0.015 (three kinases), and differences observed in Nek2 (NHW observed mean: 16.53, sd: 1.99; NHB observed mean: 17.30, sd = 1.48), yielded an effect size of 0.4. This corresponds to 99% power to detect a difference between NHB and NHW populations. Using the sample size for H/L (n = 39) with the same parameters yields a power of 49.9% suggesting that the H/L cohort in TCGA is too small to adequately assess these differences. To achieve 90% power would require 98 H/L samples. A similar TCGA analysis done with ancestry [58] revealed that the expression of *Nek2* and *TTK* is elevated in subjects with over 50% African ancestry (Fig. 2D, E). On the other hand, TBK1 is overexpressed in patients with 50% African ancestry or less (Fig. 2F).

We also performed a TCGA expression analysis of mRNA levels by self-reported race, ethnicity, and subtype (Table 2). This analysis has some rigor limitations due to the limited sample size. Nevertheless, this indicated that *TTK* and *Nek2* are overexpressed in NHB women, and *TBK1* in NHW, when all breast cancers are taken into consideration; this result closely follows the ancestry data presented in Fig. 2. *TTK* and *TBK1* are significantly overexpressed in NHW women with basal breast cancers, and *TTK* in basal and Luminal A breast cancers from H/L women. *TTK* and *Nek2* are significantly overexpressed in NHB women with breast cancers that do not fall within any specific molecular classification, or that failed to classify (other). The results indicate that there are racial, ethnic and subtype-specific patterns of mRNA expression of the centrosome-mitotic kinases *TTK*, *Nek2*, and *TBK1*.

**Table 2:** Average expression per patient (standard deviation) as a function of race (TCGA) Average expression of TTK, Nek2, and TBK1 by subtype, race, and ethnicity using the TCGA database

Subtype	Race	Number of cases	TTK levels^a^	NEK2 levels^a^	TBK1 levels^a^
All subtypes	NHB	182	0.19 (0.99)^b^	1.20 (1.78)^c^	− 0.36 (1.48)
	NHW	749	− 0.02 (1.01)	0.78 (1.63)	0.29 (1.57)^d^
	H/L	38	− 0.07 (0.88)	0.66 (1.72)	.05 (1.29)
	Non-H/L	875	0.07 (1.08)	0.9 (1.67)^d^	0.2 (1.7)
Basal	NHB	53	1.09 (0.94)	2.35 (1.8)	− 0.85 (1.09)
	NHW	106	1.6 (1.32)^b^	2.03 (1.75)	− 0.27 (1.28)^b^
	H/L	3	2.6 (0.05)^d^	2.9 (1.47)	− 0.78 (0.02)
	Non-H/L	149	1.5 (1.4)	2.2 (1.79)	− 0.42 (1.27)
Luminal A	NHB	56	− 0.51 (0.33)	0.18 (1.03)	− 0.36 (1.23)
	NHW	384	− 0.51 (0.32)	0.14 (1.04)	0.30^d^ (1.47)
	H/L	22	− 0.23 (0.44)^b^	1.74 (2.18)	0.14 (0.9)
	Non-H/L	403	− 0.51 (0.3)	2.05 (1.60)	0.22 (1.5)
Other	NHB	23	0.28 (1.14)^b^	1.5 (2.19)^b^	− 0.33 (2.35)
	NHW	71	− 0.47 (0.46)	0.06 (1.23)	− 0.15 (1.71)
	H/L	7	− 0.45 (0.41)	− 0.28 (0.4)	0.04 (1.9)
	Non-H/L	79	− 0.46 (0.80)	− 0.03 (1.63)	− 0.53 (1.86)

^a^mRNA Expression normalized to diploid samples (standard deviation of the population) from cBIOPORTAL/TCGA. A z-score threshold ± 2.0 was used for optimal results. P-values were done by T-test (2-tails, unequal variance)

^b^P ≤ 0.05

^c^P ≤ 0.005

^d^P ≤ 0.0005 from a T-test with 2-tails and unequal variance

A side-by-side comparison of mRNA levels of several mitotic regulators was done by race, using the TCGA database (Additional file 1: Fig. S1). This analysis indicates that the mRNA levels of multiple mitotic regulators, including TTK, Nek2, PLK1, Cyclin B1, BUB1, Aurora kinases A and B, and NDC80 (also known as HEC1) are elevated in breast cancers in NHB women. When analyzed by subtype, PLK1 and Aurora kinase B are significantly elevated in TNBC from NHB women, while TTK and TBK1 are significantly elevated in NHW women with TNBC. NHW women with Her2 + breast tumors significantly overexpress TTK, TBK1, Nek2, BUB1, and SGOI. The most common breast cancer subtype is Luminal A; NHB women with Luminal A breast cancers significantly overexpress PLK1, AURKB, and NDC80, while TBK1 is significantly overexpressed in Luminal A breast cancers from NHW women. AURKB is overexpressed in Luminal B breast cancers in NHB women. All mitotic regulators analyzed except TBK1 are significantly overexpressed in breast cancers that do not fall under a traditional subtype, or that failed to classify.

In addition, several transcription factors, including FOXM1, E2F1, E2F2, and E2F3, and c-Myc are known to regulate the expression of key cell cycle regulators. Therefore, we performed the same analysis as above and determined that all are significantly overexpressed in breast cancers in NHB women (Additional file 1: Fig. S2). E2F1 and c-Myc are overexpressed in basal breast cancers from NHB women, FoxM1 in Her2 + breast cancers from NHW women, FoxM1, E2F1 and E2F2 in Luminal A breast cancers from NHB women. Similarly to the patterns of expression of mitotic kinases in breast cancers that do not fall under traditional classification (or that failed to classify) FOXM1, E2F1, E2F2, E2F3, and c-Myc are overexpressed in that subtype.

Saavedra and Chellappan’s labs have demonstrated that the silencing of Nek2, TTK, or TBK1 can individually modulate rates of centrosome amplification-driven chromosome instability and aneuploidy [35, 39, 41, 59, 60]. Therefore, we performed TCGA analysis of the average aneuploidy index (0 representing no aneuploidy, and 35 maximal aneuploidy) in NHB, NHW, H/L, and Non-H/L to determine if it is significantly higher in particular subtypes, race or ethnicity and if these indexes correlate with the mRNA expression of mitotic kinases. The average aneuploidy index is significantly higher in NHB relative to NHW women in all breast cancer subtypes (Table 3). There were no differences between H/L and Non-H/L in any of the subtypes, perhaps due to the small sample size of H/L women recruited to TCGA. Notably, dysregulated levels of all mitotic kinases, including *Nek2* and *TTK* mRNAs presented in Table 2 and Additional file 1: Figure S1 correlate with increased aneuploidy in all breast cancer subtypes but only dysregulated expression of PLK1 and AURKB correlate with higher aneuploidy indexes in NHB women with basal breast cancers. This suggests that mitotic kinase overexpression may in part contribute to the overall higher aneuploidy indexes in NHB women.

**Table 3:** Average Aneuploidy indexes by race and ethnicity (Standard Deviation of the Population) from TCGA

Subtype	Race or ethnicity	Number of patients	Average aneuploidy index (SD)
All breast cancers	NHB	181	13.4 (7.8)^b^
	NHW	749	11.6 (7.8)
	H/L	33	10.4 (8.0)
	Non-H/L	806	12.2 (7.8)
Basal	NHB	53	17.6 (6.4)^a^
	NHW	106	14.63 (6.9)
	H/L	3	16.7 (4.5)
	Non-H/L	149	15.5 (7.0)
Her2 +	NHB	14	14.71 (7.9)
	NHW	37	15.9 (6.0)
	H/L	2	21 (3)
	Non-H/L	61	15.6 (6.8)
Lum A	NHB	56	8.73 (6.9)
	NHW	384	9.97 (7.7)
	H/L	17	9.6 (7.2)
	Non-H/L	403	9.7 (7.5)
Lum B	NHB	26	14.2 (6.6)
	NHW	126	15.4 (7.11)
	H/L	5	11 (6.7)
	Non-H/L	153	15.3 (7.5)
Normal	NHB	9	4.6 (8.6)
	NHW	25	6.3 (7.6)^c^
	H/L	4	0.25 (0.4)
	Non-H/L	30	8.2 (7.8)^c^
Other	NHB	23	15.6 (6.9)^c^
	NHW	71	8.3 (6.2)
	H/L	2	16 (7)
	Non-H/L	10	10.1 (5.8)

A z-score threshold ± 2.0 was used for optimal results

^a^P ≤ 0.05

^b^P ≤ 0.005

^c^P ≤ 0.0005 from a T-test with 2-tails and unequal variance

### TTK expression correlates with EMT and proliferation biomarkers

**Fig. 3:**
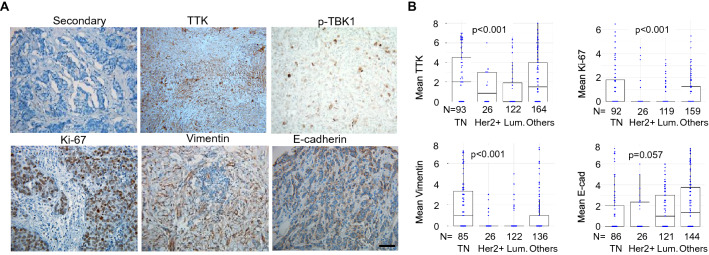
A breast cancer tissue microarray showing TTK correlates with EMT and proliferation biomarkers in different breast cancer pathological subtypes. **A**. Representative IHC images using several surrogate markers of breast cancer (40X). The scale = 50 microns. Mean Allred scores of the indicated proteins related to pathological subtypes **B**. p-TBK1 levels were assessed in TNBC patients from the USA. P-values were derived from the Kruskal–Wallis Test

The expression of several surrogate markers of poor prognosis, including TTK, TBK1, E-cadherin (low levels associated with EMT), Vimentin (high levels in mesenchymal states), and Ki67 (proliferation marker) was assessed through immunohistochemical (IHC) staining of TMAs from the NCI BMaP-3 region (now BMaP region 2) (Fig. 3A). Nek2 and pH3 IHCs were done, but the pathologist determined that the staining was not consistent between slides. This TMA is unique since it is the first microarray that contained a similar number of breast cancer patients from different ethnic and racial groups; patient characteristics, including average age, ethnicity, race, and country of origin have been published [50]. Results indicate that TTK, Ki67, and Vimentin are significantly expressed in the TNBC subtype (Fig. 3B). These results are consistent with the nature of TNBC, which has high mitotic indexes and is overrepresented among metastatic breast cancers [61, 62].

### Correlation of biomarker scores with clinical variables within the breast cancer cohort

Allred's score [63] from the TMA results for the five biomarkers TTK, TBK1, Vimentin, E-cadherin, and Ki67 were categorized as non-expression (Allred score = 0) and expression (Allred score > 0). Comparisons were done using Chi-squared tests (or Fisher’s exact test if the expected frequencies are < 5). We evaluated associations of the TTK cohort with different variables including race (NHB compared to NHW), ethnicity (Non-H/L vs. H/L), race and ethnicity (NHB, NHW, and H/L), ER, PR, Her2, and mean score of the other biomarkers (Vimentin, E-cadherin, and Ki67), Table 4. TTK expression is significantly elevated in NHW relative to NHB, in non-Hispanics (NHW and NHB) relative to H/L, and NHW relative to NHB and H/L. In addition, TTK expression is more highly overexpressed in TNBC, Her2 + , and Other subtypes relative to Luminal (ER + PR +) subtypes. TTK expression correlates with higher expression of Vimentin, E-cadherin, and Ki67.

**Table 4:** The association of TTK score with the clinical variables in the total cohort

	TTK expression = 0	TTK expression > 0	P-overall
	*N* = *214*	*N* = *191*	
Race			0.004
Black	62 (52.1%)	57 (47.9%)	
White	41 (33.1%)	83 (66.9%)	
Ethnicity			< 0.001
Non-Hispanics	103 (42.4%)	140 (57.6%)	
Hispanics	111 (68.5%)	51 (31.5%)	
Race and ethnicity			< 0.001
Non-Hispanic Black	62 (52.1%)	57 (47.9%)	
Non-Hispanic White	41 (33.1%)	83 (66.9%)	
Hispanics	111 (68.5%)	51 (31.5%)	
ER_PR_Her2			< 0.001
ER-/PR-/Her2-	35 (37.6%)	58 (62.4%)	
ER-/PR-/Her2 +	13 (50.0%)	13 (50.0%)	
ER + PR + /Her2- or +	89 (73.0%)	33 (27.0%)	
Others	77 (47.0%)	87 (53.0%)	
Mean vimentin score:			0.010
Vimentin expression = 0	158 (58.7%)	111 (41.3%)	
Vimentin expression > 0	43 (43.0%)	57 (57.0%)	
Mean E-cadherin score			0.015
E-cadherin expression = 0	120 (59.4%)	82 (40.6%)	
E-cadherin expression > 0	81 (46.3%)	94 (53.7%)	
Mean Ki67 score			0.041
Ki67 expression = 0	154 (56.4%)	119 (43.6%)	
Ki67 expression > *0*	55 (44.7%)	68 (55.3%)	

Allred score of TTK was treated as the categorical variable as non-expression (Allred score = 0, n = 214) and expression (Allred score > 0, n = 191).The clinical variables include race, hormone receptors, and mean score of Vimentin, E-cadherin, and Ki67 biomarkers. P-values were derived from Chi-squared tests

Next, we analyzed the Vimentin score with the clinical variables (Additional file 1: Table S1). The expression of Vimentin is significantly elevated in NHW patients relative to NHB patients, and NHW relative to NHB and H/L breast cancer patients. Vimentin levels are higher in TNBC relative to other breast cancer subtypes. Vimentin levels also correlated with TTK and Ki67 expression, and it was inversely correlated with the expression of E-cadherin.

Analysis of the correlation of E-cadherin with the clinical variables (Additional file 1: Table S2) does not show a correlation between E-cadherin with race. The analysis showed that E-cadherin levels are significantly higher in non-H/L relative to H/L, and in NHB and NHW when compared to H/L. There are no significant differences in the levels of E-cadherin between different breast cancer subtypes or with proliferation markers. There is a significant correlation between high E-cadherin expression with TTK expression, and it is inversely correlated with the expression of vimentin. When we analyzed the Ki67 score with the clinical variables (Additional file 1: Table S3) the expression of Ki67 is significantly higher in NHW relative to NHB, in non-H/L relative to H/L, and NHW relative to NHB and H/L. Ki-67 levels are higher in TNBC relative to other subtypes. Ki-67 significantly correlates with high TTK and Vimentin expression. We did not find a correlation with E-cadherin (P = 0.705).

We then analyzed a smaller cohort of TNBC patients from Moffitt Cancer Center (N = 129) for the expression of TTK, active (p-TBK1), Ki-67, Vimentin, and E-cadherin in NHB and NHW patients. In this smaller cohort of patients, TTK levels do not correlate with markers of EMT or proliferation (Additional file 1: Table S4). As the results presented above for a larger cohort, TTK overexpression is higher in NHW relative to NHB patients. There are no correlations between p-TBK1 expression and proliferation, or EMT markers (Table 5). Elevated Vimentin, E-cadherin, or Ki-67 levels do not correlate with levels of other surrogate markers of prognosis in this smaller cohort (Additional file 1: Tables S5, S6, S7).

**Table 5:** The association of TBK1 score with the clinical variables in the Moffitt Cancer Center’s TNBC cohort

	TBK1 expression = 0	TBK1 expression > 0	P-value
	*N* = *63*	*N* = *66*	
Race			0.182
Black	22 (59.5%)	15 (40.5%)	
White	41 (44.6%)	51 (55.4%)	
Mean TTK score			0.213
TTK expression = 0	20 (40.8%)	29 (59.2%)	
TTK expression > 0	43 (53.8%)	37 (46.2%)	
Mean Vimentin score			0.684
Vimentin expression = 0	20 (46.5%)	23 (53.5%)	
Vimentin expression > 0	29 (52.7%)	26 (47.3%)	
Mean E-cadherin score:			1.000
E-cadherin expression = 0	33 (52.4%)	30 (47.6%)	
E-cadherin expression > 0	22 (52.4%)	20 (47.6%)	
Mean Ki67 score			0.091
Ki67 expression = 0	36 (56.2%)	28 (43.8%)	
Ki67 expression > 0	25 (39.7%)	38 (60.3%)	

Allred score of TBK1 was treated as the categorical variable as non-expression (Allred score = 0, n = 63) and expression (Allred score > 0, n = 66). The clinical variables include race, hormone receptors, and mean score of TTK, E-cadherin, and Ki67 biomarkers. P-values were derived from Chi-squared tests

## Discussion

Besides their role in regulating centrosome homeostasis and mitosis, centrosome-associated mitotic kinases can drive cancer progression. Several of these kinases, including Nek2 and TTK, can drive early intermediates to metastasis, including the epithelial-to-mesenchymal transition, cell migration, and cell invasion [24, 35, 49]. Polo-like kinases and Aurora kinases showed efficacy in recent clinical trials; however, these drugs also appear to have limited efficacy in solid tumors as single agents [64–67] perhaps due to overlapping roles of mitotic kinases during mitosis [45]. Thus, mitotic-based therapies are currently been refined, and small molecule inhibitors against several centrosome and mitotic regulators are currently in clinical trials, including a phase II trial with the inhibitor ENMD-2076, which is specific against the Aurora kinase A and angiogenesis kinases, against metastatic, triple-negative breast cancers [68]. That clinical trial found that the clinical benefit rate (patients that achieved partial, complete responses or stable disease) was 16.7% in 6 months after commencing treatment, and 27.8% in 4 months after commencing treatment; the most common side effects were hypertension, fatigue, diarrhea, and nausea. None of these patients achieved complete responses. Another phase II clinical trial that focused on breast cancer patients utilized the Aurora kinase A inhibitor Alisertib along with Paclitaxel compared to Paclitaxel alone [69]. The 22 month progression-free survival was significantly reduced when Alisertib was added to Paclitaxel, while the differences in overall survival were non-significant. However, the addition of Alisertib enhanced grade 3 or 4 adverse effects, including neutropenia, sepsis, anemia, diarrhea, and stomatitis or oral mucositis. When considering the TNBC cohort only, the addition of Paclitaxel and Alisertib significantly reduced progression-free survival, while a trend toward significance was observed in overall survival (P = 0.09). Results from TTK clinical trials have recently been published, and some adverse effects (severe anemia, fatigue, and neutropenia at the highest doses) were reported with combined treatments with TTK inhibitor S81694 [70], or with Paclitaxel and TTK inhibitor BAY1217389 [71] in patients with diverse solid tumors. The results are encouraging since 32–34% of patients achieved stable disease. However, > 3 neutropenia was attributed to increases in TTK inhibitor. These results suggest that these inhibitors must be used in combination with other drugs that include microtubule agents. Some TBK1 inhibitors are FDA approved for inflammatory diseases (e.g. Amlexanox) and derivatives are been tested pre-clinically as anticancer agents [29, 72–74]. Our previous work shows that co-inhibiting the mitotic kinases TTK and TBK1 may represent a more effective way of suppressing mitotic markers such as Cyclin B expression, the phosphorylation of the mitotic markers Aurora kinases A, B, and C, pH3, and cell viability relative to the single inhibition of either kinase [31].

In the present study, we evaluated mRNA expression patterns of the mitotic kinases Nek2, Mps1/TTK, and TBK1, as well as the expression and association of these proteins with biomarkers of proliferation (Ki67) and EMT (E-cadherin and Vimentin) by using a novel breast cancer TMA. Besides its role in the cell cycle and SAC, TTK is overexpressed and correlates with advanced stages in several cancer types such as NSCLC [75, 76], prostate [77], colon [78], and breast [49, 79, 80] cancers. Previous studies analyzing TTK expression from breast cancer biopsies for each of the main breast cancer subtypes including TNBC, Her2, Luminal A, and Luminal B found that TTK overexpression was specific for the TNBC subtype [79].

NIMA-related kinase 2 (Nek2) is understudied in cancer compared with other cell cycle regulators. However, Nek2 is overexpressed in many cancers, including those of the liver, lungs, pancreas, glioma, colon, prostate, and breast [81–87]. Nek2 overexpression has been described as a prognostic biomarker for disease progression and patient survival in different cancer types, including breast cancer [87–89]. *TTK* and *Nek2* (along with VRK1, MASTL, SRPK1, CDC7, AURKA, PLK1, AURKB, CHEK1, CDC2, BUB1, MELK, PBK, BUBB1, and PLK4) belong to a 16-kinase mRNA signature that can identify basal breast cancers and a subset of poor-prognosis Luminal patients [90]. In this study, *Nek2* also showed a high overall expression in samples from breast cancer patients, which is consistent with our previous studies, as well as other studies highlighting elevated Nek2 gene expression in breast cancer, including the TNBC subtype [35, 91]. Despite the general overexpression of Nek2 and TTK shown in breast cancer, no clear information exists on whether this is a general or specific pattern related to the genetic context of patients. Limited evidence regarding the contribution of ancestry and mitotic kinase gene expression exists. A meta-analysis conducted by Wang et al. 2019, identified Nek2 as a worse prognostic predictor in solid cancers for Asians [87]. NHB and H/L are typically more severely affected by breast cancer disparities when compared with NHW [4, 92–94]. In the present study, we demonstrate that NHB had the highest gene expression for the *Nek2* and *TTK* mRNAs, as well as the mRNAs of other mitotic regulators, including *PLK1*, *CCNB1*, *BUB1*, *AURKA*, *AURKB,* and *NDC80* (also known as *HEC1*), while NHW had the higher levels of TBK1. When we analyzed the gene expression of these kinases by subtype, *TTK* mRNA is more highly overexpressed in basal breast cancers from NHW and H/L patients, and Luminal A patients from H/L. However, it is known that TNBCs have a higher prevalence in NHB and H/L compared to NHW and that more studies with proportional subtype representation are needed. PLK1 and AURKB are more highly overexpressed in basal breast cancers from NHB. All the mitotic regulators above, including *Nek2* and *TTK,* are also overexpressed in non-classified breast tumors. No explanation was given for this unclassified group in the original TCGA manuscript [54]; this group could also represent tumors that failed to classify due to technical reasons. The overexpression of *Nek2* and *TTK* also correlates with the higher aneuploidy indexes in NHB women. On the other hand, *TBK1* mRNA was significantly overexpressed in all breast tumors from NHW patients, and in NHW patients with basal, and Luminal A subtypes. However, when protein levels of TTK, and TBK1 are analyzed by immunohistochemistry, the patterns of expression differ when compared to their mRNA levels. For example, while levels of *TTK* mRNA were higher in NHB and H/L women, its protein levels were significantly elevated in NHW women and non-H/L relative to H/L. This may be due to post-transcriptional mechanisms that may lead to changes in the protein stability of these kinases. It may also be due to the mRNAs and the protein samples originating from different cohorts (the mRNA from TCGA and the protein samples from the Puerto Rico Biobank). Future studies will be conducted using a new generation TMA where the tumor samples will also be RNA and DNA seq, so the data will exactly match. We have shown that the overexpression of E2Fs alters the protein stability of several mitotic regulators, including Cyclin B1, SgoI, and BubR1 [36] and similar mechanisms may also govern the stability of TTK and TBK1. Nevertheless, our data revealed important associations between TTK expression and subtypes that associate with a poorer prognosis, including TNBC and Her2 + breast cancers. TTK overexpression also correlated with surrogate markers of poor prognosis, including EMT (higher Vimentin levels) and with a higher proliferation index. There were also correlations of Vimentin overexpression with breast cancers in NHW women, with the TNBC subtype and with Ki67, and an inverse correlation with E-cadherin. The major difference between H/L and non-H/L was the lower levels of E-cadherin marker in H/L relative to NHB and NHW women. Regarding Ki67, it was higher in NHW women and non-H/L; H/L women had the lowest proliferation index. These results may have clinical implications since they suggest that EMT in H/L may occur through different mechanisms relative to non-H/L women since E-cadherin levels are significantly higher in the former. The lower levels of Ki67 in H/L may also be of clinical significance since high proliferation indexes are highly correlated to better responses to docetaxel-based chemotherapy in ER + breast cancer patients [95].

A possible mechanism involved in the regulation of the targets described in this study could be the Forkhead box protein M1 (FOXM1), a member of the FOX family of transcription factors that plays a key role in the G2/M phase of the cell cycle, controlling proliferation and genomic stability [96–98]. TCGA analysis showed a significant increase in the relative expression of FOXM1 in All, Luminal A, and unclassified (or that failed to classify) breast cancers for NHB, and a significant increase of relative expression in Her2 for NHW (Additional file 1: Fig. S1). Among the cell cycle genes regulated by FOXM1 are Plk1, Cyclin B2, CENPF, and Nek2. Thus, future studies through this transcription factor can establish specific molecular mechanisms of action for these mitotic kinases. Another potential mechanism (non-mutually exclusive with FoxM1 dysregulation) is that the E2Fs may also contribute to the dysregulation of mitotic regulation in NHB women. Our work has shown that Nek2 and Plk4 mRNAs are directly regulated by the E2F activators [38], while others have shown that Aurora Kinase A is a transcriptional target of E2F [99]; likewise, PLK1 is an E2F target [100].

Given the significant disparities in breast cancer patients, centrosome-associated mitotic kinases could be important targets to be evaluated in the context of genetic differences and their related significant health disparities in breast cancer.

## Conclusions

In summary, we established different correlations of centrosome-associated mitotic kinases with biomarkers from the EMT in a novel breast cancer TMA with tumor samples derived from women with different ethnic backgrounds including NHB, NHW, and C-H/L. Thus, our results showed high expression of TTK, Ki67, and Vimentin in TN tumors, while low expression of E-cadherin. TTK, but not TBK1, showed significant correlations with all the clinical variables measured. On the other hand, TCGA dataset analysis revealed that the mRNA levels of several centrosome-mitotic kinases were significantly higher in NHB versus NHW women. However, the H/L results were not significant due to the limited sample size. Thus, TTK and Nek2 may be future potential targets for TNBC treatment in NHB and H/L populations.

## Supplementary Information

**Additional file 1: Figure S1.** Average expression of mitotic kinases and regulators by breast cancer subtypes in NHBs and NHWs using the TCGA database. P-values were done by T-test (2-tails and unequal variance). The * (p≤0.05) refers to significance in NHB (relative to NHW) and the ^#^ (p≤0.05) refers to significance in NHW (relative to NHB). **Figure S2.** Average expression of FoxM1, E2Fs, and Myc transcription factors by breast cancer subtypes in NHB and NHW using the TCGA database. P-values were done by T-test (2-tails, unequal variance). The * refers to significance in NHBs (relative to NHW, p≤0.05) and the # refers to significance in NHWs (relative to NHB, p≤0.05). **Table S1.** The association of Vimentin score with the clinical variables in the total cohort. **Table S2.** The association of E-cadherin score with the clinical variables in the total cohort. **Table S3.** The association of Ki67 score with the clinical variables in the total cohort. **Table S4.** The association of TTK score with the clinical variables in the Moffitt Cancer Center’s TNBC cohort. **Table S5.** The association of Vimentin score with the clinical variables in the Moffitt Cancer Center’s TNBC cohort. **Table S6.** The association of E-cadherin score with the clinical variables in the Moffitt Cancer Center’s TNBC cohort. **Table S7.** The association of Ki67 score with the clinical variables in the Moffitt Cancer Center’s TNBC cohort.

## Data Availability

All the data generated as part of this study are included in this published article and its Additional files.
