# Supplementary material for: Ethnic and racial-specific differences in levels of centrosome-associated mitotic kinases, proliferative and epithelial-to-mesenchymal markers in breast cancers
Source: Cell Div. 2022 Dec 9;17:6. doi: 10.1186/s13008-022-00082-3 (PMC9733043; doi:10.1186/s13008-022-00082-3)
Supplement: Supplementary file 1 — Additional file 1: Figure S1. Average expression of mitotic kinases and regulators by breast cancer subtypes in NHBs and NHWs using the TCGA database. P-values were done by T-test (2-tails and unequal variance). The * (p≤0.05) refers to significance in NHB (relative to NHW) and the # (p≤0.05) refers to significance in NHW (relative to NHB). Figure S2. Average expression of FoxM1, E2Fs, and Myc transcription factors by breast cancer subtypes in NHB and NHW using the TCGA database. P-values were done by T-test (2-tails, unequal variance). The * refers to significance in NHBs (relative to NHW, p≤0.05) and the # refers to significance in NHWs (relative to NHB, p≤0.05). Table S1. The association of Vimentin score with the clinical variables in the total cohort. Table S2. The association of E-cadherin score with the clinical variables in the total cohort. Table S3. The association of Ki67 score with the clinical variables in the total cohort. Table S4. The association of TTK score with the clinical variables in the Moffitt Cancer Center’s TNBC cohort. Table S5. The association of Vimentin score with the clinical variables in the Moffitt Cancer Center’s TNBC cohort. Table S6. The association of E-cadherin score with the clinical variables in the Moffitt Cancer Center’s TNBC cohort. Table S7. The association of Ki67 score with the clinical variables in the Moffitt Cancer Center’s TNBC cohort. [file 13008_2022_82_MOESM1_ESM.docx]

**
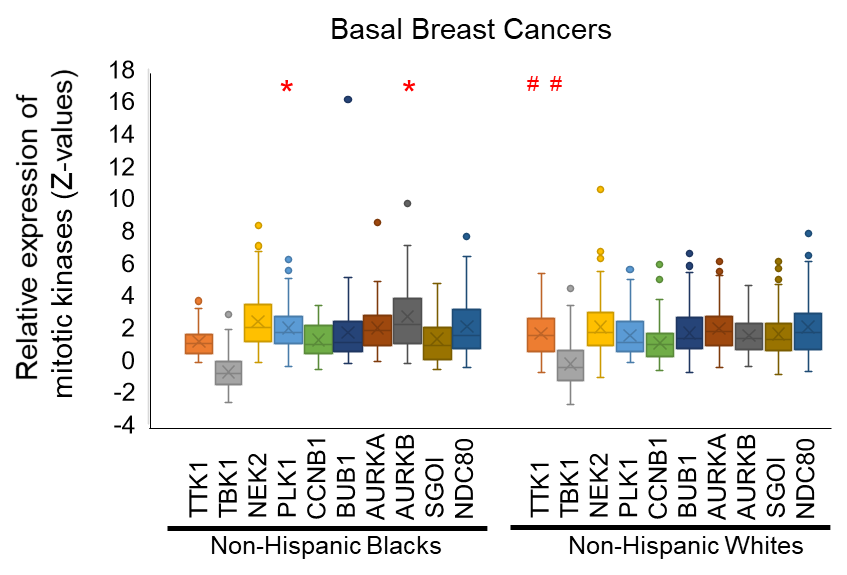

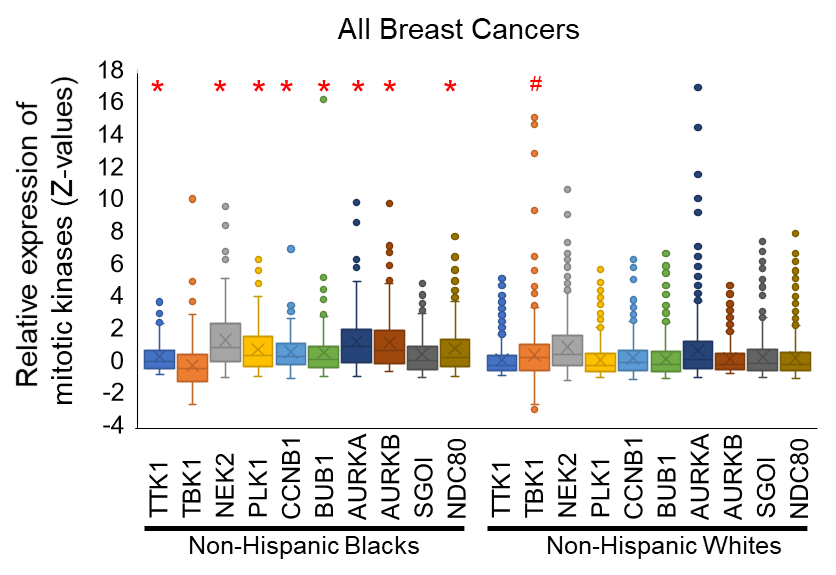
**

**A**

**B**

**C**

**
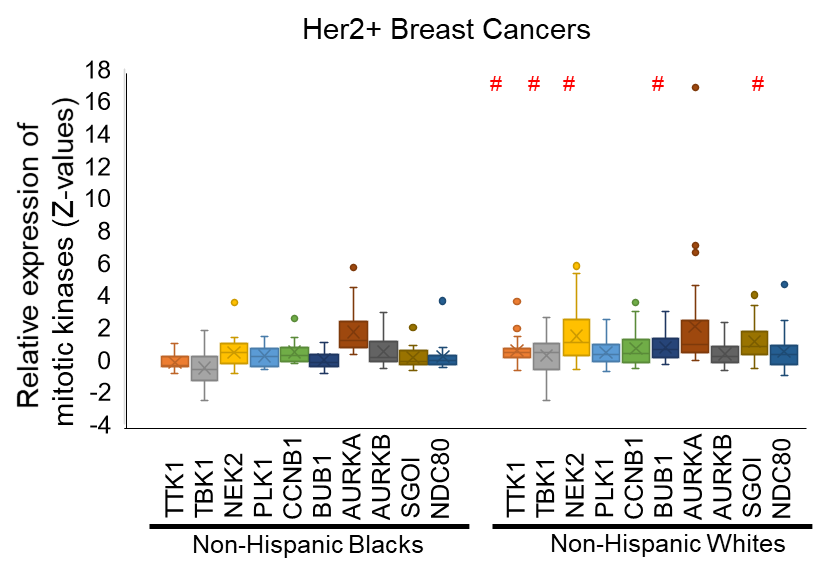
**

**
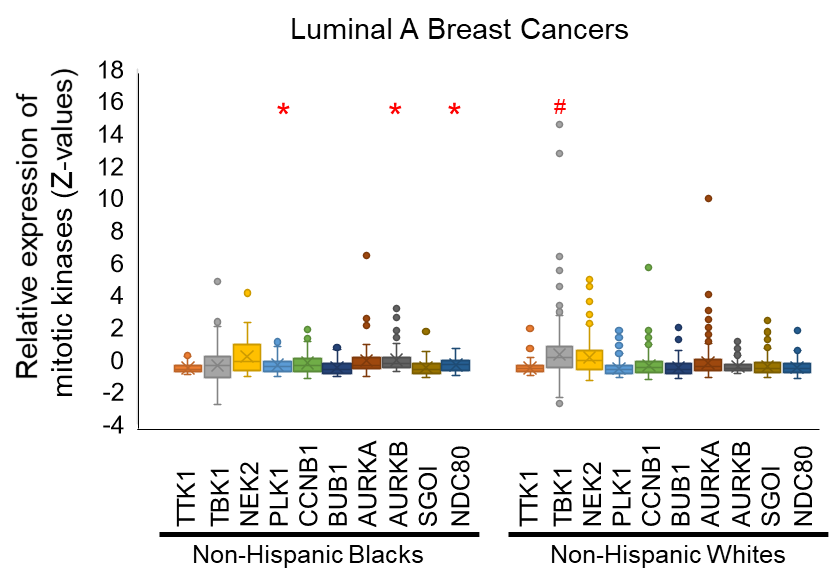
**

**F**

**E**

**D**

**
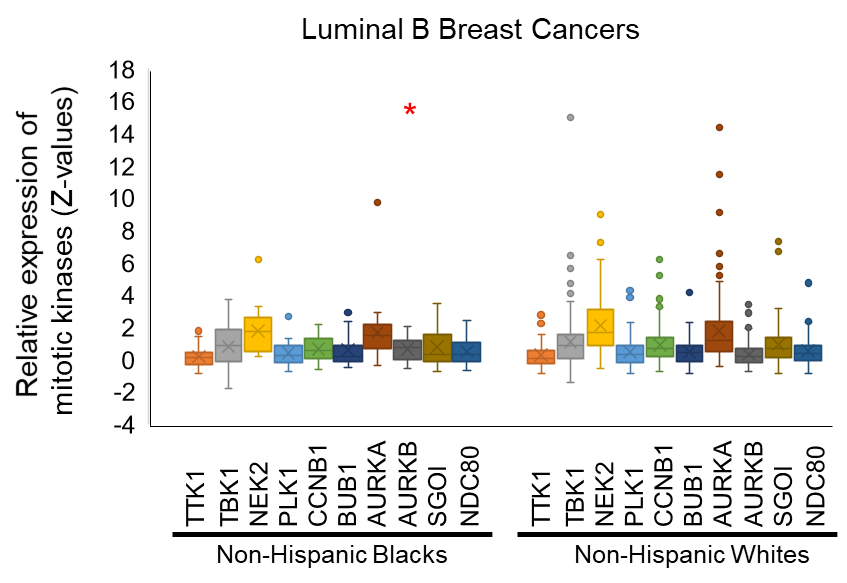

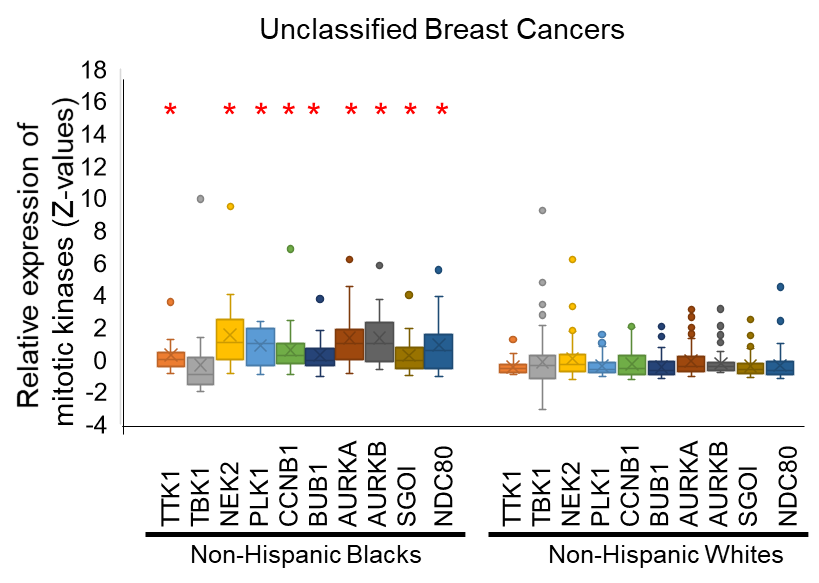
**

**Additional file 1: Figure S1. Average expression of mitotic kinases and regulators by breast cancer subtypes in NHBs and NHWs using the TCGA database.** P-values were done by T-test (2-tails and unequal variance). The * (p≤0.05) refers to significance in NHB (relative to NHW) and the ^#^ (p≤0.05) refers to significance in NHW (relative to NHB).

**
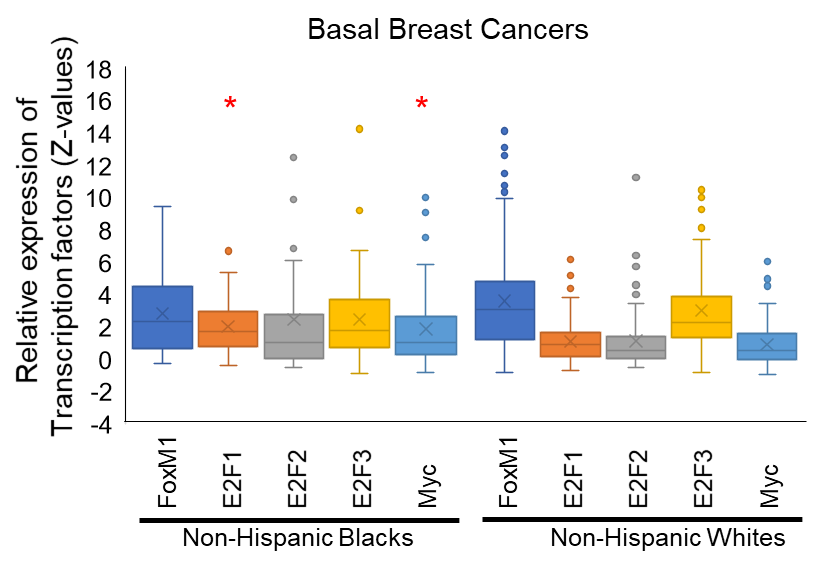
**

**D**

**C**

**B**

**A**

**
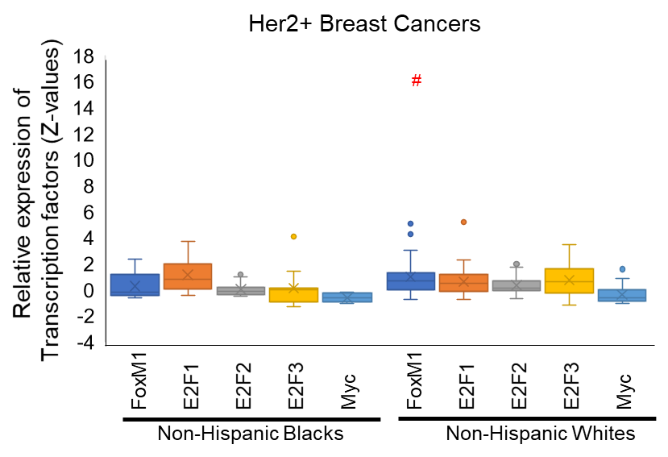

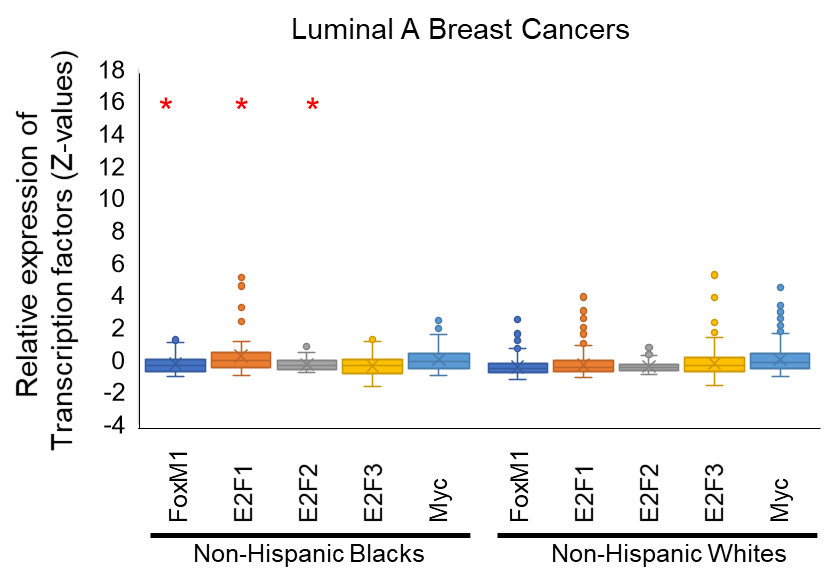

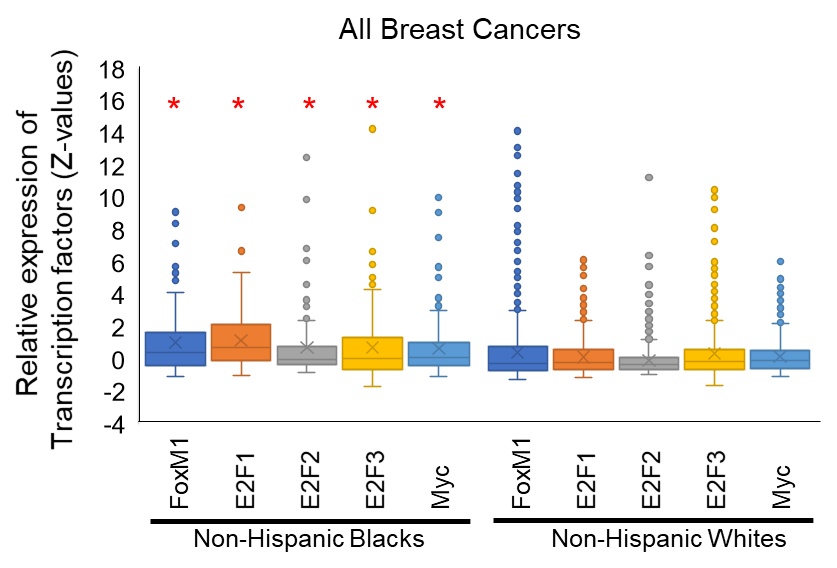
**

**
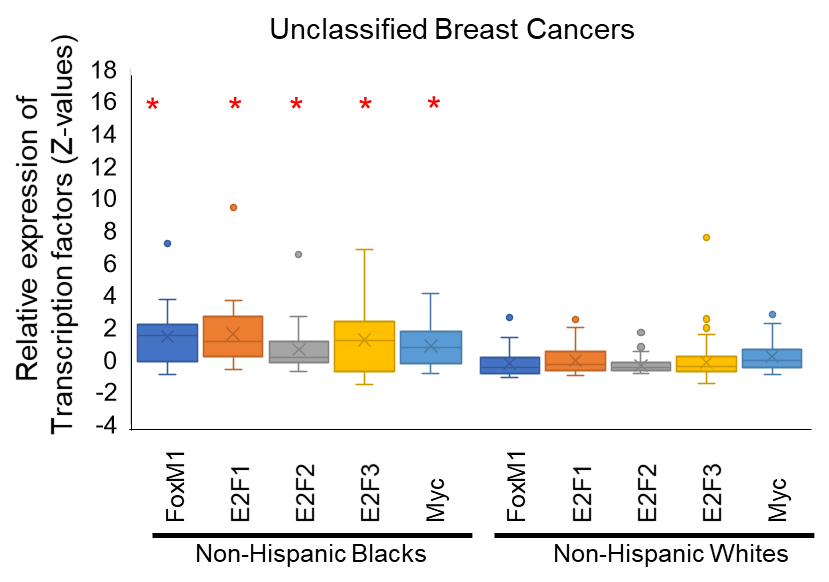
**

**E**

**F**

**
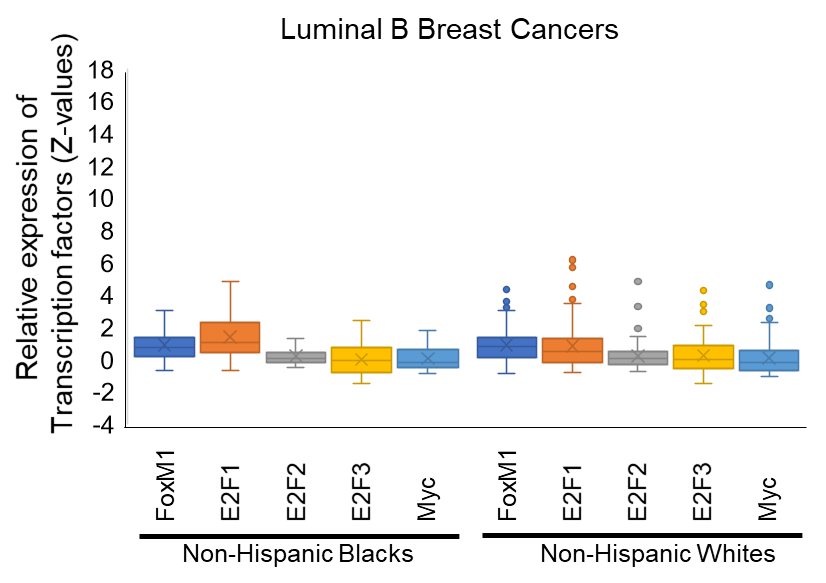
Additional file 1: Figure S2. Average expression of FoxM1, E2Fs, and Myc transcription factors by breast cancer subtypes in NHB and NHW using the TCGA database.** P-values were done by T-test (2-tails, unequal variance). The * refers to significance in NHBs (relative to NHW, p≤0.05) and the # refers to significance in NHWs (relative to NHB, p≤0.05).

**Additional file 1: Table S1. The association of Vimentin score with the clinical variables in the total cohort**

|  | **Vimentin expression = 0** | **Vimentin expression > 0** | **p-value** |
| --- | --- | --- | --- |
|  | ***N=269*** | ***N=100*** |  |
| Race: |  |  | <0.001 |
| Black | 88 (82.2%) | 19 (17.8%) |  |
| White | 55 (54.5%) | 46 (45.5%) |  |
| Ethnicity: |  |  | 0.055 |
| Non-Hispanic | 143 (68.8%) | 65 (31.2%) |  |
| Hispanics | 126 (78.3%) | 35 (21.7%) |  |
| Race/Ethnicity: |  |  | <0.001 |
| Non-Hispanic Black | 88 (82.2%) | 19 (17.8%) |  |
| Non-Hispanic White | 55 (54.5%) | 46 (45.5%) |  |
| Hispanics | 126 (78.3%) | 35 (21.7%) |  |
| ER PR Her2: |  |  | <0.001 |
| ER-/PR-/Her2- | 38 (44.7%) | 47 (55.3%) |  |
| ER-/PR-/Her2+ | 21 (80.8%) | 5 (19.2%) |  |
| ER+PR+/Her2- or + | 111 (91.0%) | 11 (9.02%) |  |
| Others | 99 (72.8%) | 37 (27.2%) |  |
| Mean TTK score: |  |  | 0.010 |
| TTK expression = 0 | 158 (78.6%) | 43 (21.4%) |  |
| TTK expression > 0 | 111 (66.1%) | 57 (33.9%) |  |
| Mean E-cadherin score: |  |  | 0.043 |
| E-cadherin expression = 0 | 138 (69.0%) | 62 (31.0%) |  |
| E-cadherin expression > 0 | 128 (79.0%) | 34 (21.0%) |  |
| Mean Ki67 score: |  |  | 0.001 |
| Ki67 expression = 0 | 200 (77.8%) | 57 (22.2%) |  |
| Ki67 expression > 0 | 65 (60.7%) | 42 (39.3%) |  |

The association of Vimentin with clinical variables. Allred score of Vimentin was treated as the categorical variable as non-expression (Allred score = 0, n = 269) and expression (Allred score > 0, n= 100). The clinical variables include race, hormone receptors, and mean score of TTK, E-cadherin, and Ki67 biomarkers. P-values were derived from Chi-squared tests.

**Additional file 1: Table S2. The association of E-cadherin score with the clinical variables in the total cohort**

|  | **E-cadherin expression = 0** | **E-cadherin expression > 0** | **p-value** |
| --- | --- | --- | --- |
|  | ***N=202*** | ***N=175*** |  |
| Race: |  |  | 0.724 |
| Black | 50 (44.2%) | 63 (55.8%) |  |
| White | 49 (47.6%) | 54 (52.4%) |  |
| Ethnicity: |  |  | 0.001 |
| Non-Hispanic | 99 (45.8%) | 117 (54.2%) |  |
| Hispanic | 103 (64.0%) | 58 (36.0%) |  |
| Race/ethnicity: |  |  | 0.002 |
| Non-Hispanic Blacks | 50 (44.2%) | 63 (55.8%) |  |
| Non-Hispanic Whites | 49 (47.6%) | 54 (52.4%) |  |
| Hispanic | 103 (64.0%) | 58 (36.0%) |  |
| ER PR Her2: |  |  | 0.058 |
| ER-/PR-/Her2- | 56 (65.1%) | 30 (34.9%) |  |
| ER-/PR-/Her2+ | 16 (61.5%) | 10 (38.5%) |  |
| ER+PR+/Her2- or + | 60 (49.6%) | 61 (50.4%) |  |
| Others | 70 (48.6%) | 74 (51.4%) |  |
| Mean TTK score: |  |  | 0.015 |
| TTK expression = 0 | 120 (59.7%) | 81 (40.3%) |  |
| TTK expression > 0 | 82 (46.6%) | 94 (53.4%) |  |
| Mean Vim score: |  |  | 0.043 |
| Vimentin expression = 0 | 138 (51.9%) | 128 (48.1%) |  |
| Vimentin expression > 0 | 62 (64.6%) | 34 (35.4%) |  |
| Mean Ki67 score: |  |  | 0.705 |
| Ki67 expression = 0 | 142 (54.6%) | 118 (45.4%) |  |
| Ki67 expression > 0 | 57 (51.8%) | 53 (48.2%) |  |

The association of E-cadherin with clinical variables. Allred score of E-cadherin was treated as the categorical variable as non-expression (Allred score = 0, n = 202) and expression (Allred score > 0, n= 175). The clinical variables include race, hormone receptors, and mean score of TTK, Vimentin, and Ki67 biomarkers. P-values were derived from Chi-squared tests.

**Additional file 1: Table S3. The association of Ki67 score with the clinical variables in the total cohort**

|  | **Ki67 expression = 0** | **Ki67 expression > 0** | **p-value** |
| --- | --- | --- | --- |
|  | ***N=273*** | ***N=123*** |  |
| Race: |  |  | 0.009 |
| Black | 81 (69.8%) | 35 (30.2%) |  |
| White | 63 (52.5%) | 57 (47.5%) |  |
| Ethnicity: |  |  | <0.001 |
| Non-Hispanic | 144 (61.0%) | 92 (39.0%) |  |
| Hispanic | 129 (80.6%) | 31 (19.4%) |  |
| Race/ethnicity: |  |  | <0.001 |
| Non-Hispanic Black | 81 (69.8%) | 35 (30.2%) |  |
| Non-Hispanic Whites | 63 (52.5%) | 57 (47.5%) |  |
| Hispanic | 129 (80.6%) | 31 (19.4%) |  |
| ER_PR_Her2: |  |  | <0.001 |
| ER-/PR-/Her2- | 53 (57.6%) | 39 (42.4%) |  |
| ER-/PR-/Her2+ | 20 (76.9%) | 6 (23.1%) |  |
| ER+PR+/Her2- or + | 99 (83.2%) | 20 (16.8%) |  |
| Others | 101 (63.5%) | 58 (36.5%) |  |
| Mean TTK score: |  |  | 0.041 |
| TTK expression = 0 | 154 (73.7%) | 55 (26.3%) |  |
| TTK expression > 0 | 119 (63.6%) | 68 (36.4%) |  |
| Mean Vim score: |  |  | 0.001 |
| Vimentin expression = 0 | 200 (75.5%) | 65 (24.5%) |  |
| Vimentin expression > 0 | 57 (57.6%) | 42 (42.4%) |  |
| Mean E-cadherin score: |  |  | 0.705 |
| E-cadherin expression = 0 | 142 (71.4%) | 57 (28.6%) |  |
| E-cadherin expression > 0 | 118 (69.0%) | 53 (31.0%) |  |

The association of Ki67 with clinical variables. Allred score of Ki67 was treated as the categorical variable as non-expression (Allred score = 0, n = 273) and expression (Allred score > 0, n= 123). The clinical variables include race, hormone receptors, and mean score of TTK, Vimentin, and E-cadherin biomarkers. P-values were derived from Chi-squared tests.

**Additional file 1: Table S4. The association of TTK score with the clinical variables in the Moffitt Cancer Center’s TNBC cohort**

|  | **TTK expression = 0** | **TTK expression > 0** | **p-value** |
| --- | --- | --- | --- |
|  | ***N=49*** | ***N=80*** |  |
| Race: |  |  | 0.029 |
| Black | 20 (54.1%) | 17 (45.9%) |  |
| White | 29 (31.5%) | 63 (68.5%) |  |
| Mean TBK score: |  |  | 0.213 |
| TBK expression = 0 | 20 (31.7%) | 43 (68.3%) |  |
| TBK expression > 0 | 29 (43.9%) | 37 (56.1%) |  |
| Mean Vimentin score: |  |  | 0.503 |
| Vimentin expression = 0 | 15 (34.9%) | 28 (65.1%) |  |
| Vimentin expression > 0 | 24 (43.6%) | 31 (56.4%) |  |
| Mean E-cadherin score: |  |  | 0.125 |
| E-cadherin expression = 0 | 27 (42.9%) | 36 (57.1%) |  |
| E-cadherin expression > 0 | 11 (26.2%) | 31 (73.8%) |  |
| Mean Ki67 score: |  |  | 0.631 |
| Ki67 expression = 0 | 26 (40.6%) | 38 (59.4%) |  |
| Ki67 expression > 0 | 22 (34.9%) | 41 (65.1%) |  |

Allred score of TTK was treated as the categorical variable as non-expression (Allred score = 0, n = 49) and expression (Allred score > 0, n= 80). The clinical variables include race and mean score of TBK1, Vimentin, E-cadherin, and Ki67 biomarkers. P-values were derived from Chi-squared tests.

**Additional file 1: Table S5. The association of Vimentin score with the clinical variables in the Moffitt Cancer Center’s TNBC cohort**

|  | ***Vimentin* expression = 0** | ***Vimentin* expression > 0** | **p value** |
| --- | --- | --- | --- |
|  | ***N=43*** | ***N=55*** |  |
| Race: |  |  | 0.584 |
| Black | 14 (50.0%) | 14 (50.0%) |  |
| White | 29 (41.4%) | 41 (58.6%) |  |
| Mean TTK score: |  |  | 0.503 |
| TTK expression = 0 | 15 (38.5%) | 24 (61.5%) |  |
| TTK expression > 0 | 28 (47.5%) | 31 (52.5%) |  |
| Mean TBK score: |  |  | 0.684 |
| TBK expression = 0 | 20 (40.8%) | 29 (59.2%) |  |
| TBK expression > 0 | 23 (46.9%) | 26 (53.1%) |  |
| Mean E-cadherin score: |  |  | 0.640 |
| E-cadherin expression = 0 | 30 (47.6%) | 33 (52.4%) |  |
| E-cadherin expression > 0 | 12 (40.0%) | 18 (60.0%) |  |
| Mean Ki67 score: |  |  | 0.076 |
| Ki67 expression = 0 | 27 (54.0%) | 23 (46.0%) |  |
| Ki67 expression > 0 | 16 (34.0%) | 31 (66.0%) |  |
|  |  |  |  |

Allred score of Vimentin was treated as the categorical variable as non-expression (Allred score = 0, n = 43) and expression (Allred score > 0, n= 55). The clinical variables include race and mean score of TTK, TBK1, E-cadherin, and Ki67 biomarkers. P-values were derived from Chi-squared tests.

**Additional file 1: Table S6. The association of E-cadherin score with the clinical variables in the Moffitt Cancer Center’s TNBC cohort**

|  | **E-cadherin expression = 0** | **E-cadherin expression > 0** | **p value** |
| --- | --- | --- | --- |
|  | ***N=63*** | ***N=42*** |  |
| Race: |  |  | 0.574 |
| Black | 21 (65.6%) | 11 (34.4%) |  |
| White | 42 (57.5%) | 31 (42.5%) |  |
| Mean TTK score: |  |  | 0.125 |
| TTK expression = 0 | 27 (71.1%) | 11 (28.9%) |  |
| TTK expression > 0 | 36 (53.7%) | 31 (46.3%) |  |
| Mean TBK score: |  |  | 1.000 |
| TBK expression = 0 | 33 (60.0%) | 22 (40.0%) |  |
| TBK expression > 0 | 30 (60.0%) | 20 (40.0%) |  |
| Mean Vimentin score: |  |  | 0.640 |
| Vimentin expression = 0 | 30 (71.4%) | 12 (28.6%) |  |
| Vimentin expression > 0 | 33 (64.7%) | 18 (35.3%) |  |
| Mean Ki67 score: |  |  | 0.376 |
| Ki67 expression = 0 | 34 (65.4%) | 18 (34.6%) |  |
| Ki67 expression > 0 | 28 (54.9%) | 23 (45.1%) |  |

The association of E-cadherin with clinical variables. Allred score of E-cadherin was treated as the categorical variable as non-expression (Allred score = 0, n = 63) and expression (Allred score > 0, n= 42). The clinical variables include race and mean score of TTK, TBK1, Vimentin, and Ki67 biomarkers. P-values were derived from Chi-squared tests.

**Additional file 1: Table S7. The association of Ki67 score with the clinical variables in the Moffitt Cancer Center’s TNBC cohort**

|  | **Ki67 expression = 0** | **Ki67 expression > 0** | **p-value** |
| --- | --- | --- | --- |
|  | ***N=64*** | ***N=63*** |  |
| Race: |  |  | 0.739 |
| Black | 20 (54.1%) | 17 (45.9%) |  |
| White | 44 (48.9%) | 46 (51.1%) |  |
| Mean TBK score: |  |  | 0.091 |
| TBK expression = 0 | 36 (59.0%) | 25 (41.0%) |  |
| TBK expression > 0 | 28 (42.4%) | 38 (57.6%) |  |
| Mean TTK score: |  |  | 0.631 |
| TTK expression = 0 | 26 (54.2%) | 22 (45.8%) |  |
| TTK expression > 0 | 38 (48.1%) | 41 (51.9%) |  |
| Mean Vimentin score: |  |  | 0.076 |
| Vimentin expression = 0 | 27 (62.8%) | 16 (37.2%) |  |
| Vimentin expression > 0 | 23 (42.6%) | 31 (57.4%) |  |
| Mean E-cadherin score: |  |  | 0.376 |
| E-cadherin expression = 0 | 34 (54.8%) | 28 (45.2%) |  |
| E-cadherin expression > 0 | 18 (43.9%) | 23 (56.1%) |  |

Allred score of Vimentin was treated as the categorical variable as non-expression (Allred score = 0, n = 64) and expression (Allred score > 0, n= 63). The clinical variables include race and mean score of TBK1, TTK, Vimentin, and E-cadherin biomarkers. P-values were derived from Chi-squared tests.
